# In Vitro Bioavailability Study of an Antiviral Compound Enisamium Iodide

**DOI:** 10.3390/scipharm86010003

**Published:** 2018-01-11

**Authors:** Eleonore Haltner-Ukomadu, Svitlana Gureyeva, Oleksii Burmaka, Andriy Goy, Lutz Mueller, Grygorii Kostyuk, Victor Margitich

**Affiliations:** 1 Across Barriers, Science Park 1, 66123 Saarbruecken, Germany; 2 Joint Stock Company Farmak, 63 Kyrylivska Street, 04080 Kiev, Ukraine; s.gureeva@farmak.ua (S.G.); a.goy@farmak.ua (A.G.); G.Kostyuk@farmak.ua (G.K.); v.margitich@farmak.ua (V.M.); 3 Dr. Regenold GmbH, Zoellinplatz 4, 79410 Badenweiler, Germany

**Keywords:** bioavailability, biopharmaceutics classification system, permeability, solubility

## Abstract

An investigation into the biopharmaceutics classification and a study of the in vitro bioavailability (permeability and solubility) of the antiviral compound enisamium iodide (4-(benzylcarbamoyl)-1-methylpyridinium iodide) were carried out. The solubility of enisamium iodide was determined in four different buffers. Apparent intestinal permeability (*P*_app_) of enisamium iodide was assessed using human colon carcinoma (Caco-2) cells at three concentrations. The solubility of enisamium iodide in four buffer solutions from pH 1.2 to 7.5 is about 60 mg/mL at 25 °C, and ranges from 130 to 150 mg/mL at 37 °C, depending on the pH. Based on these results, enisamium iodide can be classified as highly soluble. Enisamium iodide demonstrated low permeability in Caco-2 experiments in all tested concentrations of 10–100 μM with permeability coefficients between 0.2 × 10^−6^ cm s^−1^ and 0.3 × 10^−6^ cm s^−1^. These results indicate that enisamium iodide belongs to class III of the Biopharmaceutics Classification System (BCS) due to its high solubility and low permeability. The bioavailability of enisamium iodide needs to be confirmed in animal and human studies.

## 1. Introduction

Influenza is an acute respiratory illness caused by influenza A and B viruses. A single class of antiviral drugs—neuraminidase inhibitors: oseltamivir, zanamivir and peramivir—is currently recommended for treatment of influenza in adults and children. The shortage of antiviral medicines for the treatment of influenza dictates the need to develop new compounds that act on targets other than neuraminidase viral proteins.

**Figure 1:**
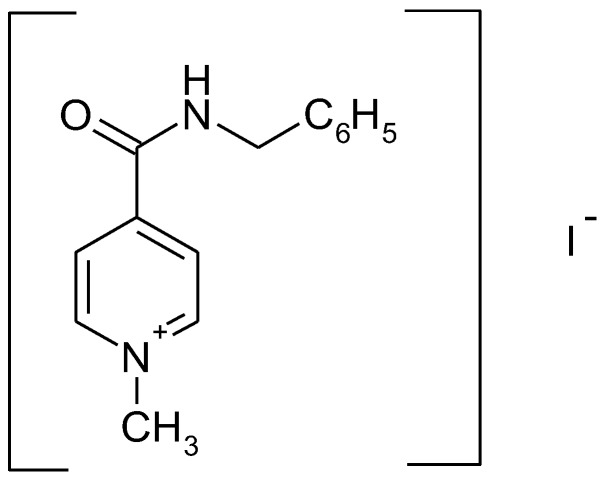
Chemical structure of enisamium iodide.

Recently enisamium iodide (IUPAC NAME: 4-(benzylcarbamoyl)-1-methylpyridinium iodide (molecular formula: C_14_H_15_IN_2_O)) (see Figure 1), a derivative of isonicotinic acid, was demonstrated to exhibit antiviral effects in vitro [1] and in vivo [2] against influenza H1N1, H3N2 and type B viruses.

Based on these findings, prediction of bioavailability of the drug substance is now warranted. For solid oral dosage, forms the bioavailability is driven by 2 steps: (1) dissolution of the drug substance from the pharmaceutical form and (2) absorption in the gastrointestinal (GI) tract. The biopharmaceutics classification system (BCS), as proposed by Amidon et al. in 1995 [3], may help to support prediction of bioavailability. The system is based on the solubility of the drug substance as well as its permeability across membranes after dissolution in the target organ system. The BSC classifies each drug substance based on solubility and permeability into the following four classes:

BCS class I “high” solubility–“high” permeability
BCS class II “low” solubility–“high” permeability
BCS class III “high” solubility–“low” permeability
BCS class IV “low” solubility–“low” permeability

According to the BCS [4], a substance is considered to be highly soluble when the highest single dose of the drug substance dissolves in less than 250 mL of buffer solution within a pH range of 1.2­–6.8 at a temperature of 37 °С. The permeability classification is based directly on the extent of intestinal absorption of a drug substance in humans or indirectly on the measurements of the rate of mass transfer across the human intestinal membrane such as human colon carcinoma (Caco-2) and calculation of the apparent permeability (*P*_app_). Low permeability is characterized by *P*_app_ values < 5 × 10^−6^ cm·s^−1^, whereas values > 5 × 10^−6^ cm·s^−1^ correspond to highly permeable drugs [5]. The BCS is a well-acknowledged tool for characterizing drug permeability and solubility/dissolution [6,7,8]. 

For investigation of the solubility of a drug substance, guidelines are available from EU and US authorities. These require demonstrating that the maximum dose strength of a drug substance can be dissolved in 250 mL of the dissolution medium within the pH range from 1 to 6.8 at 37 ± 1 °C [4,9].

An important factor in oral bioavailability is the ability of a compound to be well absorbed in the small intestine. Polarized cell monolayers are state of the art in vitro test systems that allow quick and cost-effective assessment of the permeability of a drug substance. In vitro studies using Caco-2 monolayer cells, which resemble small intestinal epithelial cells, is the gold standard to evaluate permeability of a substance [10,11,12,13,14,15,16,17,18,19]. Moreover, Caco-2 cells are the recommended testing system to determine whether a substance has low, medium, or high permeability according to international requirements [20,21,22,23,24,25].

The present study was carried out in order to classify enisamium iodide according to the BCS guidelines, based on the compound’s in vitro solubility and permeability in the Caco-2 cell system, and to predict the potential bioavailability of enisamium iodide in vivo.

## 2. Materials and Methods

### 2.1. Materials

#### 2.1.1. Solubility Study

The solubility study was conducted using enisamium iodide (CAS number 201349-37-3), which was synthesized at Farmak JSC (batches 170215, 180215 and 190315) (Kiev, Ukraine). All batches were in compliance with the in-house specification. The total content of related substances in each batch was less than 0.05%.

For preparing the buffer solutions (pH 1.2, 4.5, 6.8 and 7.5) of the solubility study the following reagents were used: hydrochloric acid fuming (purity 37.0–38.0%, Merck, KGaA, Darmstadt, Germany), acetic acid (purity 99.8–100.5%, Sigma-Aldrich, Steinheim, Germany), sodium chloride (purity ≥ 99.8%, Sigma-Aldrich), sodium hydroxide (purity 99.0–100.5%, Sigma-Aldrich), sodium acetate (purity ≥ 99.0%, Sigma-Aldrich), and potassium phosphate monobasic (purity 98.0–100.5%, Sigma-Aldrich). 

#### 2.1.2. Permeability Study

Enisamium iodide and *N*-methyl-4-*N*-ethanolpyridinium iodide (internal standard for LC-MS/MS) were provided by Farmak JSC.

Dulbecco’s Modified Eagle’s Medium (DMEM) was obtained from Pan Biotech (Aidenbach, Germany). [^3^H]-propranolol, rhodamine 123, Krebs-Ringer buffer (KRB), cyclosporine A and [^3^H]-digoxin from Sigma-Aldrich. Trypsin/ethylenediaminetetraacetic acid (EDTA) solution and sodium dodecyl sulfate from Merck (Darmstadt, Germany). Fluorescein was purchased from VWR International (Darmstadt, Germany). Culture flasks were obtained from VWR International and Transwell filter inserts from Corning Life Sciences (Lowell, MA, USA).

### 2.2. Methods

#### 2.2.1. Solubility Study

Solubility was investigated in four different buffer systems at two temperatures each. Additionally, the solubility of enisamium iodide in buffer solution pH 7.5 was performed according to recommendations described at Food and Drug Administration (FDA) Guidance for Industry [26].

Preparation of buffer solutions:

pH 1.2: 425.0 mL of 0.2 M hydrochloric acid solution, and 250.0 mL 0.2 M sodium chloride solution diluted to 1000 mL with water;

pH 4.5: 2.99 g of sodium acetate, 14 mL of 2 M acetic acid solution diluted to 1000 mL with water;

pH 6.8: 250.0 mL of 0.2 M monobasic potassium phosphate solution, 112.0 mL of 0.1 M sodium hydroxide solution diluted to 1000 mL with water;

pH 7.5: 250.0 mL of 0.2 M monobasic potassium phosphate solution, 112.0 mL of 0.1 M sodium hydroxide solution diluted to 1000 mL with water and adjusted to pH 7.5 with 0.1 M sodium hydroxide solution and.

Preparation of stock test solution: To 40 mL of the buffer solution, an excess amount of enisamium iodide (2.9 g in the study at 25 °C and 6.9 g in the study at 37 °C) was added. The flask was tightly closed, placed on a shaking incubator (GFL, model 3031, Burgwedel, Germany), and agitated at 140 rpm for 96 h (48 h for buffer pH 1.2 at 37 °C).

The solubility experiments were performed at 25 °C and 37 °C.

Determination of enisamium iodide was carried out by an HPLC method using a Zorbax Eclipse XDB column (C_18_, 4.6 mm × 150 mm, 5 µm particle size, Agilent Technologies, Amstelveen, The Netherlands). Mobile phase: buffer solution pH 2.5:acetonitrile:purified water (26:30:44 *v*/*v*/*v*); Preparation of buffer solution pH 2.5: a mixture of 1.085 g of sodium octane-1-sulfonate monohydrate and 1.000 g disodium hydrogen phosphate anhydrous was dissolved in 900 mL of water, adjusted with phosphoric acid to pH 2.5 ± 0.05 and diluted with water to 1000 mL. Flow rate of the mobile phase: 0.5 mL min^−1^ and a column temperature of 30 °C. The samples were injected at a volume of 10 µL with detection at wavelength 225 nm.

Preparation of the final test solution: Following stirring, the flask was allowed to deposit the excess amount of substance for 5 min. Thereafter, 1.0 mL of the supernatant was transferred into a 50 mL volumetric flask, dissolved in mobile phase, and adjusted to 50.0 mL with the same solvent. For the study at 25 °C, 5.0 mL of this solution was diluted to 20.0 mL with mobile phase. For the study at 37 °C, 5.0 mL of the solution was diluted to 50.0 mL with mobile phase.

Preparation of reference solution: 400.0 mg of the reference standard of enisamium iodide was dissolved in mobile phase and diluted with the same solvent to 100.0 mL. 10.0 mL of this solution was dissolved to 100.0 mL with mobile phase.

#### 2.2.2. Permeability Study

Cell culture. For the maintenance culture, Caco-2 cells were incubated at 37 °C, 5% CO_2_, and 90% relative humidity in 75 cm^2^ culture flasks with supplemented Dulbecco’s Modified Eagle’s Medium (DMEM). The cells were passaged once a week using trypsin/ethylenediaminetetraacetic acid (EDTA) solution. Approximately 10^6^ cells were seeded per flask. The culture medium was changed three times a week.

For the transport studies, the Caco-2 cells were inoculated at a density of 60,000 cells/cm^2^ on Transwell filter inserts and placed into 12-well flat-bottom cluster plates. The inserts (apical compartments) were supplied with 0.5 mL and the outer wells (basal compartments) with 1.5 mL of DMEM culture medium. The cells for the present transport study were cultured at 37 °C, 5% CO_2_ and 90% relative humidity in DMEM culture medium for 18 days until they formed confluent monolayers (31 passages). The culture medium was replaced every 2–3 days. Cell monolayers with a trans-epithelial electrical resistance (TEER) of not less than 200 Ω·cm^2^ were used.

Tolerability of enisamium iodide to Caco-2 cells. For this purpose, the cells were incubated with the highest dose supposed for the permeability studies (100 μM). After an incubation period of 120 min at 37 °C the TEER was checked to assess the integrity of the cell monolayers. The applied enisamium iodide solution was replaced by the growth medium to analyze the reversibility of the cell monolayer’s integrity within 24 h. All experiments were performed in triplicate. As a positive control, sodium dodecyl sulfate was used in this experiment.

Transport studies. Caco-2 monolayers were rinsed twice with KRB to remove the cell culture medium. Fresh KRB (pH 7.4) was filled into the acceptor compartment and the transport solution (test substance in KRB at pH 7.4) into the donor compartment. The cells were equilibrated for 30 min in a CO_2_ incubator at 37 °C and stayed there between the sample collection points. After pre-incubation, samples were taken from the donor and acceptor compartment, respectively. The concentration of test compound in the donor compartment was defined as initial donor concentration. At 0 min, 30 min, 60 min, 90 min and 120 min 100 µL samples were drawn from the acceptor compartment. The amount of solution removed was replaced by fresh, pre-warmed KRB. At the final pull point, samples were retrieved from the donor compartment, too. All tests were carried out in triplicate. For studies including the P-glycoprotein (P-gp) inhibitor cyclosporin A and the marker compound for P-gp transport inhibitors, [^3^H]-digoxin the substances were applied to both the donor and acceptor compartment in the same concentration. The intactness of the monolayer cells was verified during the experiments by measuring the TEER at the beginning and end with a limit of not less than 200 Ω·cm^2^.

Testing of integrity of monolayers. Each batch of monolayer Caco-2 cells was inoculated and cultured under the same conditions on Transwell filter inserts in parallel. Qualification of intactness of the Caco-2 monolayer batches prior to the transport studies was carried out in triplicate for each transport condition using the selected transport markers fluorescein, [^3^H]-propranolol and rhodamine 123.

The transport of enisamium iodide in apical-to-basolateral (ab) direction was investigated at concentrations of 10 µmol·L^−1^, 50 µmol·L^−1^, and 100 µmol·L^−1^. The influence of the presence of a specific P-gp inhibitor was carried out using cyclosporin A at a concentration of 12 µmol·L^−1^ on the ab as well as the basolateral-to-apical (ba) transport of enisamium iodide (10 µmol·L^−1^). The samples of enisamium iodide were analyzed by LC-MS/MS. In order to evaluate the potential of enisamium iodide (10 µmol·L^−1^) to act as a P-gp inhibitor, its effects on the bi-directional transport (ab as well as ba) of [^3^H]-digoxin at a concentration of 1 µmol·L^−1^ was determined. The apical side with a slightly acidic pH represents the average pH in the lumen of the small intestine, and the basolateral side with neutral pH simulates the pH of the blood.

Determination of enisamium iodide was carried out by an LC-MS method using a Atlantis column (C_18_, 4.6 mm × 150 mm, 5 µm particle size, Waters, Milford, MA, USA). Mobile phase A consisted of 0.1% formic acid in water and mobile phase B of 0.1% formic acid in acetonitrile. A nine-minute gradient elution (0.00 to 1.00 min 95% phase A, 1.00 to 5.00 min 95–10% phase A, 5.00 to 5.10 min 10–95% phase A and 5.10 min to end 95% phase A) was used with a constant flow rate of 1.2 mL·min^−1^ and a column temperature of 40 °C. The samples were kept at 10 °C and injected at a volume of 20 µL. The triple quad mass spectrometer system (Waters) was used in the positive (ESI^+^) electrospray mode at 2.63 kV. Quantification was carried out for the multiple reaction monitoring (MRM) transition m/z 227.18 to 94.02 (enisamium iodide) and 180.96 to 134.92 (internal standard *N*-methyl-4-*N*-ethanolpyridinium iodide).

Fluorescein and rhodamine 123 samples (100 µL) were determined by fluorescence reading. The samples were collected in 96 well microtiter plates. The fluorescence intensity was recorded by exciting the sample with light at 485 nm and measuring the emitted fluorescence at 535 nm. The fluorescence emission of a blank sample (Krebs–Ringer bicarbonate buffer (KRB) solution) was used as baseline.

[^3^H]-propranolol and [^3^H]-digoxin were quantified by liquid scintillation counting. The samples (300 μL) were transferred into 24 well plates and mixed with 500 μL liquid scintillation cocktail. After an equilibration period of at least 1 h radiation was measured for 2 min per well in a scintillation counter. The recorded counts in a blank sample were subtracted from the value of each test sample.

Data of the permeability study were calculated using MS Excel (Microsoft, Redmond, WA, USA). Apparent permeability coefficient (*P*_app_) was calculated according to the following equation:

$$P_{\mathrm{app}}=\frac{\Delta Q\;1\;1}{\Delta t\;m_{0}\;A}\;V_{D}\left(\mathrm{cm}\cdot s^{-1}\right)$$

where Δ*Q*/Δ*t* is the permeability rate (steady state transport rate) obtained from the profile of the transported mass or radioactivity of substrate versus time (µg or dpm·s^−1^). Calculated by the linear regression of time and concentration. *A* is the area of the exposed cell monolayer (cm^2)^, *m*_0_ is the initial mass or radioactivity of test compound in the donor compartment (µg or dpm), and *V*_*D*_ is the buffer volume of donor compartment. 

The TEER was calculated according to

$$\mathrm{TEER}={\;R}_{c\left(A\right)}=\left(R_{c+f}-R_{f}\right)A\;\left(\Omega \cdot {\mathrm{cm}}^{2}\right).$$

where *R_c_*_(A)_ is the electrical resistance of the monolayer with the area *A* (Ω cm^2^); *R*_c+f_ is the electrical resistance of the monolayer including the filter (Ω); *R*_f_ is the electrical resistance of the filter without cells (Ω); and *A* is the area of the monolayer (cm^2^).

The efflux ratio is calculated as

(1) $$\mathrm{Efflux}\;\mathrm{ratio}=\;\frac{P_{\mathrm{app}}\;\left(\mathrm{BA}\right)}{P_{\mathrm{app}}\;\left(\mathrm{AB}\right)}$$

where *P*_app_ (ba) is the apparent permeability coefficient for the transport of the test substance from the basal to the apical side (secretive direction) and *P*_app_ (ab) is the apparent permeability coefficient for the transport of the test substance from the apical to the basal side (absorptive transport).

## 3. Results

### 3.1. Solubility Study

Table 1 shows the results of the solubility study of enisamium iodide in mg/mL in four buffer solutions at two temperature conditions. No significant difference between the batches tested was observed.

### 3.2. Permeability Study

The Caco-2 monolayer batch used for the experiments met all acceptance criteria. It was demonstrated by the transport of fluorescein that the monolayers represented a tight barrier for low permeable compounds and that propranolol as compound known for high (*P*_app_ (AB) = 19.58 × 10^−6^ cm·s^−1^) and fluorescein known for low permeability (*P*_app_ (AB) = 0.18 × 10^−6^ cm·s^−1^) could clearly be discriminated.

The integrity and robustness of the monolayers were emphasized by the TEER values before and after the transport of the qualification markers (values for all transporter experiments were between 384 and 610 Ω·cm^2^). Furthermore, the expression and functionality of the P-gp efflux system were shown by the high secretion of rhodamine 123 (*P*_app_ (BA) = 4.50 × 10^−6^ cm s^−1^; *P*_app_ (BA)/*P*_app_ (AB) = 17.09).

The investigation of the tolerability of Caco-2 cells on the test substance showed that none of the tested concentrations (up to 100 μM enisamium iodide) had an impact on the TEER of the Caco-2 cells. After 2 h of incubation, the TEER remained at about 100% of the TEER measured in medium without test substance. Positive control with sodium dodecyl sulfate, a well know substance impacting the integrity of Caco-2 cells [27], showed immediate and remaining reduction of TEER to <108 Ω·cm^2^. After recovery, the TEER was determined to be only 118 Ω·cm^2^.This demonstrated that the test system was valid.

**Figure 2:**
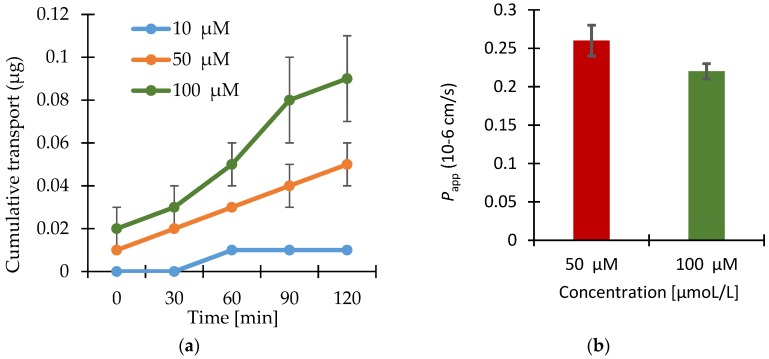
(**a**) Apical to basolateral transport of enisamium iodide at three different concentrations through Caco-2 cell monolayers. Shown values are the individual values from each Transwell^®^ insert and the relevant arithmetic means value (*n* = 3) ± standard deviation (SD). The cumulative transport into the acceptor compartment is expressed in μg per 1.13 cm^2^ cell monolayer; (**b**) Apparent permeability coefficients for the apical to basolateral transport of enisamium iodide through Caco-2 cell monolayers calculated using the data points indicated in Figure 2a. Data shown are arithmetic mean values (*n* = 3) ± SD. *P*_app_ for 10 µM was not calculated since there is no steady-state flux.

**Figure 3:**
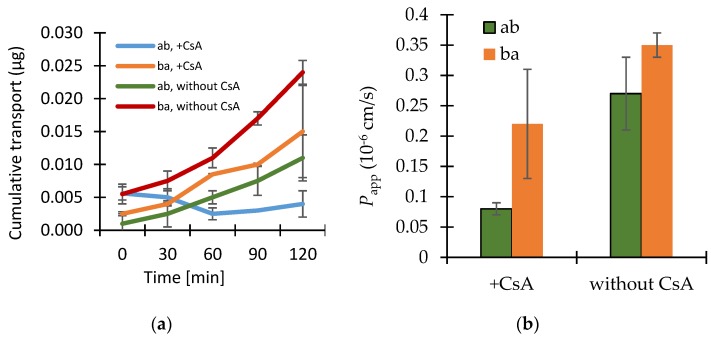
(**a**) Bi-directional cumulative transport of enisamium iodide in the presence or absence of the P-glycoprotein (P-gp) inhibitor cyclosporin A (CsA, 12 μmol·L^−1^) through Caco-2 cell monolayers. Data shown are arithmetic mean values (*n* = 3) ± SD, except the data from ba transport without cyclosporin A as there was one outlier (data performed in parallel only). (**b**) The cumulative transport for the bi-directional transport of enisamium iodide (10 μmol·L^−1^) in the presence or absence of the P-gp inhibitor cyclosporin A (CsA, 12 μmol·L^−1^) through Caco-2 cell monolayers. Data shown are arithmetic mean values (*n* = 3) ± SD, except the data from ba transport without cyclosporin A as there was one outlier (data performed in parallel only).

**Figure 4:**
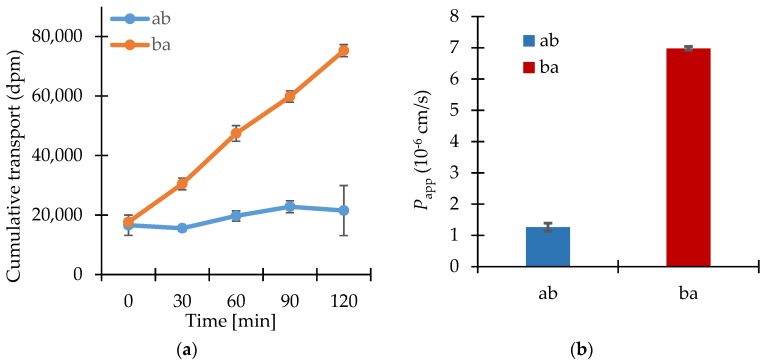
(**a**) Bi-directional cumulative transport of [^3^H]-digoxin (1 μmol·L^−1^) in the presence of the putative P-gp inhibitor enisamium iodide (10 μmol·L^−1^) through Caco-2 cell monolayers. Data shown are arithmetic mean values (*n* = 3) ± SD. (b) Apparent permeability coefficients for the bi-directional transport of [^3^H]-digoxin (1 μmol·L^−1^) in the presence of enisamium iodide (10 μmol·L^−1^) through Caco-2 cell monolayers. Data shown are arithmetic mean values (*n* = 3) ± SD.

The results of the transport studies with enisamium iodide in different concentrations, with inhibitor P-gp-Cyclosporin A (CsA) and with enisamium iodide itself as potential inhibitor of the mentioned transporter are shown in Figure 2, Figure 3 and Figure 4.

The transport of enisamium iodide is dependent on the concentration applied on the cell monolayer’s surface. Higher amounts applied in the donor compartment resulted in higher cumulative transport amounts (see Figure 2).

The permeability coefficient varied between 0.27 × 10^−6^ cm·s^−1^ and 0.22 × 10^−6^ cm·s^−1^ for the transport of enisamium iodide in concentrations between 10–100 μM. The results provide no evidence for active transport of enisamium iodide and indicate dose linearity during the transport experiments.

The P-gp inhibitor cyclosporin A at a concentration of 12 μmol·L^−1^ caused a more than 3 times lower permeation coefficient for the transport of enisamium iodide (10 μM) in the ab direction compared to without CsA (Figure 3). 

In presence of the test substance enisamium iodide at 10 μmol·L^−1^, [^3^H]-digoxin was transported much more in the ba direction as compared to the ab (indicating active efflux transport of digoxin) (Figure 4).

## 4. Discussion

The solubility found for enisamium iodide indicates that amounts of at least 31.8 g can be dissolved in 250 mL of buffer solution at 37 °C. Clinical doses have yet to be established for enisamium iodide, but since it is highly unlikely that doses of 31 g per day will have to be used in therapeutic use, the drug substance meets the criteria of a highly soluble drug substance.

The monolayer batch of Caco-2 cells used in the studies met all quality criteria. It was demonstrated by the transport of fluorescein that the monolayers represented a tight barrier for low permeable compounds, which are mainly transported via the paracellular route. Additionally, compounds featuring high (propranolol) and low permeability (fluorescein) could clearly be discriminated.

The integrity and robustness of the monolayers were emphasized by the steady TEER values before and after the transport of the qualification markers. Furthermore, the expression and functionality of the P-gp efflux system was shown by the high secretion of rhodamine 123.

The absorption of a test compound is characterized by ranking its calculated *P*_app_ into permeability classes based on *P*_app_ values of model drugs. The classification into low and high permeability drugs may be influenced by carrier systems like P-glycoprotein. In comparison with published data, enisamium iodide is estimated to have low in vitro permeability, which suggests a low intestinal absorption in vivo.

The presence of the P-gp inhibitor cyclosporin A in a concentration of 12 μmol·L^−1^ resulted in a more than three times lower permeation coefficient for the transport of enisamium iodide (10 μM) in an ab direction, which is not indicative for a P-gp-mediated transport of enisamium iodide. Nevertheless, there seems to be an interaction between cyclosporin A and enisamium iodide, which could not be clarified by this experiment. As both drugs would not be administrated at the same time (contrary indications) the drug-drug interaction is not relevant in clinical use.

Enisamium iodide did not inhibit the transport of [^3^H]-digoxin, a P-gp substrate, so it can be concluded that an interaction with P-gp is not probable.

## 5. Conclusions

Enisamium iodide demonstrated low permeability in Caco-2 experiments in all tested donor concentrations of 10–100 μM with permeability coefficients between 0.2 × 10^−6^ cm s^−1^ and 0.3 × 10^−6^ cm s^−1^.

The solubility of enisamium iodide at 25 °C is about 60 mg/mL in all four-buffer solutions, and from 130 mg/mL to 150 mg/mL at 37 °C depending on the pH buffer solution. Despite the fact that the clinical dose has still to be established, according to the BCS the substance enisamium iodide can be characterized as highly soluble.

Thus, it can be concluded that enisamium iodide can be considered as a representative of a class III compound according to the BCS due to its high solubility and low permeability.

## Figures and Tables

**Table 1:** Solubility of enisamium iodide in different standard media (mg/mL). Results for relative standard deviation are based on three separate experiments.

Batch No.	pH 1.2		pH 4.5		pH 6.8		pH 7.5	
	25 °C	37 °C	25 °C	37 °C	25 °C	37 °C	25 °C	37 °C
170215	63.2	151.5	59.7	132.2	60.8	131.3	58.9	126.7
180215	63.5	155.0	59.3	132.7	59.5	128.0	59.2	129.9
190315	63.2	154.7	60.9	135.5	60.2	131.4	59.1	125.8
Mean	63.3	153.7	60.0	133.5	60.2	130.3	59.1	127.5
RSD %	0.22	1.26	1.34	1.32	1.07	1.47	0.27	1.70

RSD: Relative standard deviation.
